# Validation of self-reported height and weight in a large, nationwide cohort of U.S. adults

**DOI:** 10.1371/journal.pone.0231229

**Published:** 2020-04-13

**Authors:** James M. Hodge, Roma Shah, Marjorie L. McCullough, Susan M. Gapstur, Alpa V. Patel

**Affiliations:** Behavioral and Epidemiology Research Group, American Cancer Society, Atlanta, GA; Cincinnati Children's, UNITED STATES

## Abstract

**Background:**

Height and weight are commonly used metrics in epidemiologic studies to calculate body mass index. Large cohort studies generally assess height and weight by self-report rather than by measurement. The aim of this study was to assess the validity of self-reported height and weight in the Cancer Prevention Study-3 (CPS-3), a large, nationwide cohort recruited by the American Cancer Society between 2006–2013.

**Methods:**

In a subset of CPS-3 participants (n = 2,643), weight and height were assessed at the same time via self-report and in-person measurement. BMI was calculated and classified underweight (<18.5 kg/m^2^), normal (18.5-<25 kg/m^2^), overweight (25-<30 kg/m^2^), or obese (≥30 kg/m^2^). Self-reported and measured height, weight, and BMI were compared using mean differences and Bland-Altman plots and examined by sex, race/ethnicity, education, marital status, age group, and BMI category.

**Results:**

Men and women slightly overreported height and underreported weight. BMI calculated from self-reported data was lower than for measured data for men and women. In analyses stratified by race/ethnicity, age, education, and marital status, older women and women with less than a college degree overreported height. Approximately 13% of men and 7% of women were misclassified into a lower self-reported BMI category, with misclassification of BMI being greatest in obese men and women.

**Conclusions:**

Overall, height, weight, and BMI were well-reported, and this study further suggests that BMI computed from self-reported weight and height is a valid measure in men and women across different socio-demographic groups.

## Introduction

Excess body fatness is an important risk factor for cancer,[1] cardiovascular,[2] and all-cause mortality.[3,4] Height and weight are commonly used metrics in epidemiologic studies to calculate body mass index (BMI, kg/m^2^) as a proxy measure for excess body fatness. In large prospective cohort studies, height and weight data are frequently self-reported on surveys due to ease of collection and relatively low cost. Given the high prevalence of obesity among U.S. adults (39.8%)[5] and the importance of accurately assessing the impact of obesity on disease risk, understanding potential misclassification of BMI due to differences between self-reported and measured height and weight is essential.[6]

It is well established that anthropometric measures are subject to systematic reporting biases that may lead to differences between self-reported and measured height and weight.[6,7] BMI calculated from self-reported weight and height (self-reported BMI) is generally lower than BMI calculated from measured weight and height (measured BMI) due to the underestimation of weight and overestimation of height.[6–17] This discrepancy results in misclassification when categorizing BMI that could bias associations between BMI and chronic disease or mortality risk.[18,19] Reporting error is influenced by both physical factors and sociodemographic characteristics. Height is consistently overreported, particularly among shorter men and older men and women.[6,10,11,14,16,20,21] Weight is generally underreported by both sexes, usually to a greater extent in women and heavier individuals.[6,7,12,15,17,22] There is additional evidence to suggest that race,[14,23,24] education level,[9,25] and marital status[25] contribute to reporting error but findings are inconsistent.

While there is ample literature on the validity of self-reported height and weight across a variety of populations, there are few studies within US-based prospective cohorts enrolled in the 21^st^ century. The American Cancer Society’s Cancer Prevention Study-3 (CPS-3) is a large US-based prospective cohort study in which self-reported weight and height can be compared to measured weight and height among a diverse sub-sample of men and women. This study provides an opportunity to assess the validity of these self-reported anthropometric measures by age, marital status, education level, and race/ethnicity separately by sex in such a cohort.

## Methods

### Study population and data collection

The CPS-3 cohort is described in detail elsewhere;[26] briefly, between 2006 and 2013, 296,450 CPS-3 volunteer participants aged 30 to 65 years old enrolled in-person at community enrollment sites where they completed a self-administered enrollment survey, had their waist circumference measured, and provided a small blood sample. Respondents were asked “What is your height?” in feet and inches and “What is your current weight?” in pounds. At select sites (n = 21 sites, 2,643 participants), following completion of the enrollment survey, all participants’ height and weight were measured by a certified biometrics technician from Quest Diagnostics, Inc. using standard operating procedures. Participants’ height without shoes on was measured to the nearest inch using a stadiometer. Weight was measured to the nearest pound using a digital scale (SECA North America, Chino, CA). Height was converted to centimeters and weight was converted to kilograms for analysis. Self-reported and measured BMI were classified into World Health Organization (WHO) categories: underweight (<18.5 kg/m^2^), normal (18.5-<25 kg/m^2^), overweight (25-<30 kg/m^2^) and obese (≥30 kg/m^2^).[27] The Emory University Institutional Review Board approves all aspects of CPS-3 (#IRB00059007) and all participants provide written consent.

We excluded men and women who were missing either self-reported (n = 15) or measured (n = 80) height or weight. An additional 9 men and 10 women were excluded due to implausible differences between self-reported and measured height (≥8 inches). A total of 2,529 CPS-3 participants (712 men, 1,817 women) were included for analyses. This study is sufficiently powered (>80%) to detect differences of approximately 1cm and 1.5kg or less for all strata included in the analysis.

Prior to analysis, the top and bottom 1% of the distributions of height difference and weight difference (n = 88) were evaluated for systematic errors. It was determined that the survey scanning process had resulted in character recognition errors for 31 values that were manually corrected. An additional 50 randomly sampled subjects had their values for self-reported height and weight checked against the scanned survey images and no further errors were identified.

### Statistical analysis

Means and standard deviations of self-reported and measured height, weight, and BMI were calculated along with mean differences to evaluate the accuracy of self-reported measures. Bland Altman plots [28] with 95% limits of agreement (LOA) were constructed to assess the agreement between self-reported and measured height, weight and BMI. These plots show the difference between the self-reported and measured values over the average of the two measures. The LOA are computed as the mean difference ±1.96 SD and represent the extent of underreporting and overreporting of self-reported compared to measured values. Data were analyzed separately for men and women and stratified by age group (<40; 40–49; ≥50 years), race (non-Hispanic white; Black/African American; Hispanic; Other), education level (<4-year college; 4-year college; graduate degree), marital status (married/living with partner; separated, divorced or widowed; never married), sex-specific quartiles of measured height and weight, and BMI category (<18.5; 18.5-<25; 25-<30; ≥30 kg/m^2^). Differences among strata were assessed using ANOVA or Welch’s test when there was heterogenous variance.[29] All analyses were conducted using R version 3.5.2.

## Results

The demographic distribution of the analytic population is presented in Table 1. A higher proportion of women were <40 years at enrollment and self-reported white race/ethnicity than men. A lower proportion of women had a college or graduate degree and were married as compared to men. A greater proportion of men were classified as overweight or obese.

**Table 1:** Sociodemographic characteristics at enrollment of Cancer Prevention Study 3 participants with measured and reported height and weight, by gender.

Sociodemographic Characteristic	Men N (%)	Women N (%)
**n**	712	1,817
**Age group (years)**		
<40	179 (25.1)	512 (28.2)
40-<50	227 (31.9)	541 (29.8)
> = 50	306 (43.0)	764 (42.0)
**Race**		
White, Non-Hispanic	450 (63.2)	1,451 (79.9)
African American	165 (23.2)	201 (11.1)
Hispanic	37 (5.2)	79 (4.3)
Other/Missing	60 (8.4)	86 (4.7)
**Education**		
<4-year college	156 (21.9)	575 (31.6)
4-year college	285 (40.0)	685 (37.7)
Graduate Degree	271 (38.1)	551 (30.3)
Missing	0 (0.0)	6 (0.3)
**Marital Status**		
Married/Living with Partner	559 (78.5)	1,271 (70.0)
Separated, Divorced or Widowed	69 (9.7)	317 (17.4)
Never Been Married	84 (11.8)	227 (12.5)
Missing	0 (0.0)	2 (0.1)
**Reported BMI (kg/m^2^)**		
<18.5	0 (0.0)	28 (1.5)
18.5-<25	179 (25.1)	775 (42.7)
25-<30	317 (44.5)	505 (27.8)
30-<35	147 (20.6)	292 (16.1)
≥35	69 (9.7)	217 (11.9)
**Measured BMI (kg/m^2^)**		
<18.5	0 (0.0)	28 (1.5)
18.5-<25	142 (19.9)	724 (39.8)
25-<30	318 (44.7)	535 (29.4)
30-<35	164 (23.0)	285 (15.7)
≥35	88 (12.4)	245 (13.5)
**Measured Height quartiles (cm)**		
Q1 (Men: <173; Women: <160)	104 (14.6)	305 (16.8)
Q2 (Men: 173–178; Women: 161–165)	174 (24.4)	522 (28.7)
Q3 (Men: 179–183; Women: 166–170)	187 (26.3)	519 (28.6)
Q4 (Men: >183; Women: >170)	247 (34.7)	471 (25.9)
**Measured Weight quartiles (kg)**		
Q1 (Men: <80.9; Women: <62.3)	172 (24.2)	449 (24.7)
Q2 (Men: 80.9–90.4; Women: 62.3–71.8)	181 (25.4)	455 (25.0)
Q3 (Men: 90.5–102.3; Women: 71.9–84.8)	178 (25.0)	459 (25.3)
Q4 (Men: >102.3; Women: >84.8)	181 (25.4)	454 (25.0)

Table 2 displays the overall means and mean differences for each measure. On average, men overreported their height by 0.48 centimeters, underreported their weight by -1.54 kilograms and under reported their BMI by -0.64 kg/m^2^. Women overreported their height by 0.16 centimeters, under reported their weight by -0.88 kilograms and underreported their BMI by -0.38 kg/m^2^.

**Table 2:** Self-reported and measured mean, mean difference, and Pearson correlation coefficient for height, weight, and body mass index.

	Self-reported Mean (SD)	Measured Mean (SD)	Mean Difference (95% CI)	Pearson Correlation Coefficient
**Men**				
Height (cm)	179.27 (7.03)	178.8 (7.09)	0.48 (0.31, 0.65)	0.95
Weight (kg)	91.59 (17.58)	93.14 (18.06)	-1.55 (-1.81, -1.3)	0.98
BMI (kg/m^2^)	28.38 (4.9)	29.02 (5.11)	-0.64 (-0.74, -0.54)	0.97
**Women**				
Height (cm)	165.07 (6.73)	164.91 (6.55)	0.16 (0.07, 0.25)	0.95
Weight (kg)	74.57 (18.59)	75.44 (18.92)	-0.88 (-0.99, -0.77)	0.99
BMI (kg/m^2^)	27.28 (6.44)	27.66 (6.64)	-0.38 (-0.44, -0.33)	0.99

In stratified analyses, there were few significant differences (Table 3). Men in the lowest height quartile overreported height to a greater degree than taller men. Heavier men and those in the highest BMI category underreported their weight more compared to men who weigh less. Significant underreporting of BMI is greater among men in the lowest height quartile and men in the highest weight and BMI categories. No significant differences were observed by age, race, education, or marital status.

**Table 3:** Mean difference for reported and measured height, weight, and BMI by sociodemographic characteristics.

	Men (n = 721)			Women (n = 1 827)		
	Mean Height Difference [95% CI]	Mean Weight Difference [95% CI]	Mean BMI Difference [95% CI]	Mean Height Difference [95% CI]	Mean Weight Difference [95% CI]	Mean BMI Difference [95% CI]
**Age group (years)**						
<40	0.47 (0.14, 0.79)	-1.45 (-1.9, -0.99)	-0.61 (-0.8, -0.43)	0.02 (-0.16, 0.21)	-0.71 (-0.91, -0.5)	-0.27 (-0.36, -0.17)
40-<50	0.34 (0.02, 0.65)	-1.65 (-2.1, -1.21)	-0.63 (-0.8, -0.46)	0.1 (-0.06, 0.26)	-0.97 (-1.19, -0.75)	-0.4 (-0.5, -0.3)
> = 50	0.59 (0.34, 0.84)	-1.51 (-1.91, -1.1)	-0.66 (-0.81, -0.51)	0.29 (0.15, 0.43)	-0.93 (-1.08, -0.78)	-0.45 (-0.53, -0.38)
p-value^a^	0.4434	0.8085	0.9210	0.0498	0.1390	0.0133
**Race**						
White, Non-Hispanic	0.47 (0.26, 0.69)	-1.4 (-1.7, -1.09)	-0.59 (-0.7, -0.47)	0.17 (0.07, 0.27)	-0.86 (-0.98, -0.74)	-0.38 (-0.44, -0.33)
Black/African American	0.34 (0.01, 0.67)	-1.76 (-2.33, -1.2)	-0.68 (-0.89, -0.46)	0.19 (-0.11, 0.49)	-1.19 (-1.55, -0.84)	-0.5 (-0.67, -0.32)
Hispanic	0.14 (-0.62, 0.89)	-1.67 (-2.66, -0.68)	-0.61 (-1.05, -0.17)	0.1 (-0.36, 0.55)	-0.72 (-1.32, -0.13)	-0.32 (-0.6, -0.04)
Other/Missing	1.1 (0.56, 1.64)	-1.9 (-2.89, -0.91)	-0.95 (-1.32, -0.59)	-0.03 (-0.49, 0.43)	-0.58 (-0.95, -0.21)	-0.2 (-0.42, 0.02)
p-value	0.1133	0.5278	0.2322	0.8230	0.1218	0.2340
**Education**						
<4-year college	0.85 (0.43, 1.27)	-1.41 (-2.18, -0.64)	-0.72 (-1, -0.45)	0.37 (0.2, 0.54)	-0.92 (-1.13, -0.7)	-0.47 (-0.58, -0.37)
4-year college	0.37 (0.11, 0.62)	-1.42 (-1.77, -1.07)	-0.56 (-0.69, -0.42)	0.11 (-0.03, 0.25)	-0.85 (-1.03, -0.68)	-0.35 (-0.44, -0.27)
Graduate Degree	0.38 (0.14, 0.63)	-1.74 (-2.06, -1.41)	-0.68 (-0.81, -0.54)	-0.01 (-0.18, 0.15)	-0.88 (-1.05, -0.71)	-0.33 (-0.41, -0.24)
p-value^a^	0.1278^	0.4756	0.3609^	0.0088	0.9565	0.1903^
**Marital Status**						
Married/Living with Partner	0.5 (0.3, 0.69)	-1.54 (-1.82, -1.27)	-0.65 (-0.75, -0.54)	0.16 (0.05, 0.27)	-0.84 (-0.97, -0.71)	-0.37 (-0.43, -0.31)
Separated, Divorced or Widowed	0.55 (0.11, 0.99)	-1.49 (-2.36, -0.63)	-0.63 (-0.91, -0.34)	0.32 (0.1, 0.54)	-0.98 (-1.24, -0.71)	-0.47 (-0.6, -0.34)
Never Been Married	0.3 (-0.22, 0.83)	-1.55 (-2.38, -0.71)	-0.59 (-0.93, -0.25)	-0.1 (-0.39, 0.18)	-0.98 (-1.25, -0.7)	-0.33 (-0.47, -0.18)
p-value^a^	0.7384	0.9934	0.9292	0.0908	0.7148	0.3777
**Measured BMI (kg/m**^**2**^**)**						
<18.5	-	-	-	-0.91 (-1.44, -0.37)	0.06 (-0.21, 0.34)	0.22 (0.06, 0.38)
18.5-<25	0.04 (-0.35, 0.42)	-0.37 (-0.75, 0)	-0.13 (-0.26, 0.01)	-0.11 (-0.25, 0.03)	-0.45 (-0.56, -0.34)	-0.13 (-0.18, -0.08)
25-<30	0.54 (0.3, 0.79)	-1.35 (-1.58, -1.12)	-0.58 (-0.68, -0.48)	0.32 (0.15, 0.49)	-0.93 (-1.17, -0.69)	-0.43 (-0.54, -0.33)
> = 30	0.65 (0.36, 0.93)	-2.47 (-3.07, -1.86)	-1 (-1.22, -0.79)	0.41 (0.23, 0.59)	-1.46 (-1.68, -1.24)	-0.72 (-0.84, -0.6)
p-value^a^	0.0299	<0.0001^	<0.0001^	<0.0001^	<0.0001^	<0.0001^
**Measured Height Quartiles (cm)**^b^						
Q1	1.03 (0.64, 1.42)	-1.57 (-2.04, -1.1)	-0.89 (-1.08, -0.7)	0.44 (0.24, 0.64)	-0.84 (-1.07, -0.62)	-0.51 (-0.63, -0.38)
Q2	0.93 (0.59, 1.28)	-1.58 (-1.93, -1.22)	-0.82 (-0.99, -0.66)	0.17 (0, 0.34)	-0.62 (-0.83, -0.4)	-0.31 (-0.41, -0.21)
Q3	0.34 (0.04, 0.64)	-1.43 (-2, -0.86)	-0.56 (-0.77, -0.35)	0.05 (-0.12, 0.23)	-1.1 (-1.32, -0.88)	-0.42 (-0.52, -0.32)
Q4	0.03 (-0.27, 0.33)	-1.62 (-2.12, -1.11)	-0.47 (-0.64, -0.3)	0.08 (-0.11, 0.26)	-0.95 (-1.13, -0.77)	-0.35 (-0.44, -0.26)
p-value^a^	<0.0001	0.9559	0.0064	0.0402	0.0079	0.0641
**Measured Weight Quartiles (kg)**^c^						
Q1	0.64 (0.31, 0.96)	-0.84 (-1.19, -0.49)	-0.46 (-0.61, -0.32)	-0.17 (-0.35, 0.01)	-0.19 (-0.32, -0.06)	-0.03 (-0.1, 0.03)
Q2	0.35 (0.03, 0.67)	-1.11 (-1.42, -0.81)	-0.47 (-0.6, -0.33)	0.12 (-0.06, 0.29)	-0.82 (-0.95, -0.69)	-0.34 (-0.41, -0.27)
Q3	0.44 (0.11, 0.77)	-1.35 (-1.67, -1.04)	-0.56 (-0.7, -0.41)	0.2 (0.02, 0.39)	-0.99 (-1.27, -0.7)	-0.42 (-0.54, -0.3)
Q4	0.49 (0.13, 0.85)	-2.83 (-3.63, -2.03)	-1.06 (-1.34, -0.77)	0.48 (0.28, 0.67)	-1.51 (-1.75, -1.26)	-0.74 (-0.87, -0.61)
p-value^a^	0.6974	0.0001^	0.0021^	<0.0001^	<0.0001^	<0.0001^

^a^p-values for ANOVA except where indicated with ^ in which case they are from Welch’s test.

^b^Height quartiles: Men: <173, 173–178, 179-<183, ≥183; Women: <160, 161-<166, 166-<170, ≥170

^c^Weight quartiles: Men: <80.9, 80.9-<90.5, 90.5-<102.3, ≥102.3; Women: <62.3, 62.3-<71.8, 71.8-<84.8, ≥84.8

Women ≥50 years overreported height to a greater degree than younger women, as did women with less than a 4-year college degree compared to women with more education. Women in the lowest height quartile and highest weight quartile also overreported height more than taller and lower-weight women. Women in the highest weight quartile and the highest BMI category underreported weight and BMI more than lower-weight women. BMI calculated from self-reported height and weight is more accurate among younger women than older women. No significant differences by race or marital status were observed.

**Fig 1:**
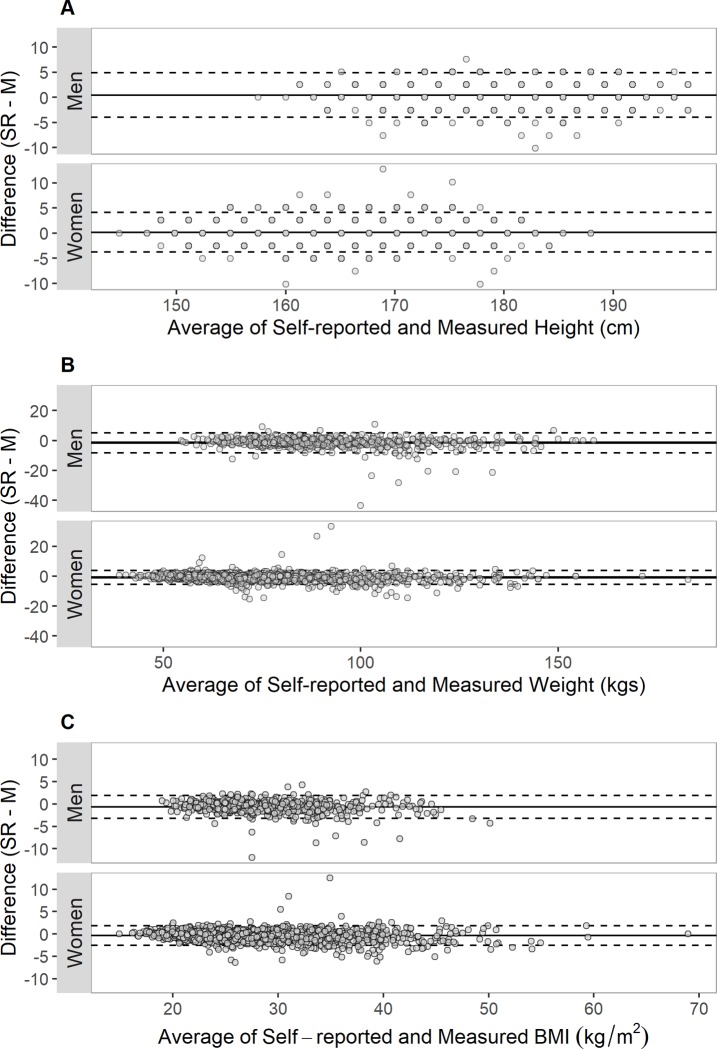
Bland-Altman plots for A) height, B) weight, and C) BMI. The solid line indicates the mean difference, the dashed lines indicate the limits of agreement (mean difference ± 1.96 times the standard deviation).

Bland-Altman plots of the difference between the two measures over their mean indicate good agreement (Fig 1). The LOA were -4.0cm to 4.9cm for men and -3.8cm to 4.1cm for women for height; -8.3kg to 5.2kg for men and -5.4kg to 3.7kg for women for weight; and -3.2kg/m^2^ to 1.9kg/m^2^ for men and -2.6kg/m^2^ to 1.8kg/m^2^ for women for BMI.

Self-reported and measured BMI categories are cross-tabulated in Table 4. The diagonals among the categories indicate the participants who were categorized in the same WHO classification based on both self-reported and measured BMI. Overall, approximately 15% of men and 10% of women had misclassified BMI. Most of the misclassification was one category lower with 15.4% of overweight men misclassified as normal and 15.5% of obese men misclassified as overweight. Results for women were similar with 14.2% of overweight women misclassified as normal and 8.5% of obese women misclassified as overweight. For men and women, a small proportion were misclassified to a higher BMI category.

**Table 4:** Cross-classification of self-reported vs. measured body mass index (kg/m^2^) categories by sex, n (%).

	Measured body mass index (kg/m^2^)			
Reported body mass index (kg/m^2^)	<18.5	18.5-<25	25-<30	≥30
**Men**				
<18.5	-	0 (0.0)	0 (0.0)	0 (0.0)
18.5-<25	-	128 (90.1)	49 (15.4)	2 (0.8)
25-<30	-	14 (9.9)	264 (83.0)	39 (15.5)
> = 30	-	0 (0.0)	5 (1.6)	211 (83.7)
**Women**				
<18.5	23 (82.1)	5 (0.7)	0 (0.0)	0 (0.0)
18.5-<25	5 (17.9)	694 (95.9)	76 (14.2)	0 (0.0)
25-<30	0 (0.0)	25 (3.5)	435 (81.3)	45 (8.5)
> = 30	0 (0.0)	0 (0.0)	24 (4.5)	485 (91.5)

## Discussion

In this subpopulation of participants in the nationwide CPS-3 cohort, there was good overall agreement between self-reported and measured height, weight, and BMI. However, in stratified analyses, we found that heavier men and women tended to underreport weight to a greater extent than lower-weight individuals, and thus self-reported BMI was lower than that for measured BMI in these individuals. Regardless, there was only modest misclassification of BMI.

Our results are generally consistent with observed reporting errors for height, weight, and BMI from previous validation studies. In a review by Connor Gorber and colleagues,[6] height was overestimated in 11 of 12 population studies among men (range of average difference: 0.5 to 2.3 cm) and 10 of 12 population studies among women (range: 0.4 to 2.2 cm). Weight was underestimated among men in 11 of 15 studies (range: -0.1 to -3.2 kg) and 13 of 14 studies among women (range: -0.3 to -3.3 kg) and BMI was consequently underestimated among both men (range: -0.3 to -2.0 kg/m^2^) and women (range: -0.2 to -2.2 kg/m^2^). Similar findings were described in a subsequent review by Maukonen and colleagues. [7] Our overall results for men and women are within the range of differences for each measure. Further, shorter individuals tend to overreport their height and heavier individuals tend to underreport their weight.[10,11,16] Our findings are consistent with this trend.

Interestingly, men underestimated their weight to a greater degree than women which is atypical but not unprecedented.[10,16] This finding is likely explained by the higher proportion of men than women in the highest BMI category (35% compared to 29%). Since obese men and women tend to underreport weight more than normal weight individuals, the greater proportion of obese men compared to women may explain difference in the mean weight difference. Nevertheless, the absolute difference for most participants was relatively small and potentially due to day-to-day variation[30] or partly attributable to weight being measured while participants were clothed while self-weighing is typically done with few clothes on.[31] Both men and women with BMI ≥30 kg/m^2^ underreported weight to a greater degree than leaner participants. This finding is similar to that observed in other studies and results in greater downward misclassification among people with higher BMI.[6,7,21,22] However, even among obese participants, the absolute mean difference in BMI remains small (-1 for men; -0.7 for women).

Age is consistently associated with reporting errors for height [10,11,14,16,20,21] with older men and women overestimating height compared to younger men and women.[10,11,14,16] We observed a similar pattern among women but not men. This result is most likely explained by the age range of our cohort with a maximum age of 65 years at enrollment. While our highest age group was ≥50, similar studies had highest age categories of ≥60 or higher. Moreover, physiological changes that may lead to overreporting height begin earlier in life in women than in men which may explain why we observed significant overreporting among older women but not older men in this study.[32] While race, education, and marital status, have also been associated with errors in self-reported height and weight, the direction and magnitude is inconsistent. [9,14,23–25] In this study, a significant difference was only seen for height by education among women.

Due to errors in self-reported height and weight, using self-reported BMI results in some misclassification when assigning BMI categories which can lead to biased risk estimates.[18] Previous studies have found misclassification ranging from 12% to approximately 20%. For example, in a population of 5,445 men and 1,905 women, Niedhammer and colleagues[9] found that 12.7% of men and 14.4% of women were misclassified. In a population of 1,870 men and 2,938 women, Spencer and colleagues[10] found 22.4% misclassification among men and 15.2% among women, using higher cut-points for low BMI (<20.0 kg/m^2^). The extent of misclassification in the current study compares favorably with these previous reports with 15% of men and 10% of women classified in a different WHO category when using self-reported BMI compared to measured BMI.

This analysis has several limitations. First, the population is a convenience sample of the larger CPS-3 population. However, these results are likely generalizable to the entire study population since most demographic characteristics were similar with the exception that this sub-population was more racially diverse and slightly younger than the full cohort.[26] Second, the height and weight measurements were taken at a variety of enrollment sites with different biometric technicians which may have introduced measurement error. Strengths of this study include the racial/ethnic diversity, inclusion of both men and women, and broad age range which allowed for various sub-group analyses. Further, it is one of few validation studies conducted in a large US-based prospective cohort including both men and women.

## Conclusions

Our findings suggest that self-reported weight and height in the CPS-3 study are subject to similar reporting errors seen consistently in prior validation studies though the absolute differences remain small. Likewise, self-reported BMI results in misclassification to a similar degree. However, our findings also demonstrate that participants report their height and weight with reasonable accuracy suggesting that BMI derived from self-reported height and weight is a valid measure across a range of socio-demographic characteristics.

## List of Abbreviations

ACS: American Cancer Society
ANOVA: Analysis of Variance
BMI: Body Mass Index
CPS-3: Cancer Prevention Study 3
LOA: Limits of Agreement
WHO: World Health Organization
